# One additional shot of brachial plexus block equates to less postoperative pain for younger children with elbow surgeries

**DOI:** 10.1186/s13018-020-01778-4

**Published:** 2020-07-06

**Authors:** Jin Li, Saroj Rai, Ruikang Liu, Ruijing Xu, Pan Hong

**Affiliations:** 1 Department of Orthopaedic Surgery, Union Hospital, Tongji Medical College, Huazhong University of Science and Technology, Wuhan, 430022 China; 2 Department of Orthopaedics and Trauma Surgery, National Trauma Center, National Academy of Medical Sciences, Mahankal, Kathmandu, Nepal; 3 First School of Clinical Medicine, Tongji Medical College, Huazhong University of Science and Technology, Wuhan, China

**Keywords:** Brachial plexus block, General anesthesia, Postoperative pain, Children

## Abstract

**Background:**

Postoperative pain in children has always been inadequately evaluated. This study aims to evaluate the postoperative pain response using an additional dose of brachial plexus block (BPB) for younger children receiving elbow surgeries under general anesthesia (GA).

**Methods:**

This retrospective case-control study included pediatric patients (3–10 years) who underwent surgeries for elbow injuries between January 2015 and January 2019. Patients with previous history of surgeries around the elbow, neurological impairment of injured limb, polytrauma, undergoing pain management for different causes, and open or old fractures were excluded. Patients were dichotomized into the GA group and the GA + BPB group as per the presence or absence of BPB.

**Results:**

In all, 150 patients (102/48, male/female) in the GA and 150 patients (104/46, male/female) in the GA + BPB group were included. There existed no significant differences between the two groups in age, sex, fracture side, and types of elbow procedures. As for the pain response after lateral condyle fracture of the humerus (LCFH), the FLACC pain scale was significantly higher for those in the GA group (6.2 ± 0.8) when compared to the GA + BPB group (1.6 ± 0.5) (*P* < 0.001). As for the pain response after medial epicondyle fracture of the humerus (MCFH), the FLACC pain scale was significantly higher for those in the GA group (6.0 ± 0.8) when compared to the GA + BPB group (1.5 ± 0.5) (*P* < 0.001). As for the pain response after supracondylar fracture of the humerus (SCFH), the FLACC pain scale was significantly higher for those in the GA group (6.0 ± 0.8) when compared to the GA + BPB group (1.6 ± 0.5) (*P* < 0.001). As for the pain response after cubitus varus correction, the FLACC pain scale was significantly higher for those in the GA group (6.7 ± 0.7) when compared to the GA + BPB group (2.1 ± 0.7) (*P* < 0.001).

**Conclusion:**

An additional shot of BPB for patients undergoing surgeries for elbow surgeries resulted in better postoperative pain response in younger children without significant BPB-related complications.

## Background

Most of the orthopedic surgeries in the pediatric population, especially in younger children, are performed under general anesthesia (GA) [1]. Age less than 10 years is considered as a young child at our institute, and usually, GA is chosen as the anesthetic modality for such patients. However, postoperative pain in children has always been inadequately evaluated [2, 3]. Healthcare workers (HCWs) in the postanesthesia care unit (PACU) and residents on night shift always complain about the difficulties tackling with crying and screaming kids in pain [4, 5]. However, pain management is influenced by many factors, such as cultural values, religions, parental beliefs, and anxiety [6, 7]. Besides, the description of pain provided by the children is usually inconclusive [8]. Several non-pharmacological methods, including position adjustment, reassurance [9], and music, have been proposed to alleviate the pain [10–12].

In order to cope with postoperative pain, additional ultrasonography (US)-guided brachial plexus block (BPB) was implemented for pediatric patients under GA since 2017 at our institute. This study aims to compare the postoperative pain response under GA with or without BPB retrospectively.

## Methods

From January 2015 to January 2019, all patients who underwent elbow surgeries under GA were retrospectively reviewed. Since 2017, additional BPB was performed in younger children at our institute, and it was consented by the parents. The results are summarized according to the type of fractures that the patient had: lateral condylar fracture of the humerus (LCFH), medial epicondyle fracture of the humerus (MCFH), supracondylar fracture of the humerus (SCFH), and cubitus varus deformity.

Inclusion criteria: (1) pediatric patients aged between 3 and 10 years who underwent surgeries for LCFH, MCFH, SCFH, and corrective osteotomy and fixation for cubitus varus deformity; (2) no previous history of surgeries around the same elbow; and (3) patients without neurological impairment. Exclusion criteria: (1) patients with polytrauma or open fractures, (2) patients with the underlying disease requiring regular pain management, (3) patients not having clear and complete medical records, and (4) delayed presentation of elbow fractures.

The pain response at PACU after extubation was evaluated by the anesthetic nurse using Face, Legs, Activity, Cry, and Consolability (FLACC) pain scale [13]. Pain response in the ward on the first night after surgery was reported by the patient using the Faces Pain Scale-Revised (FPS-R) [14], by the caregiver using the numeric rating scale (NRS) [15], and by the on-call nurse using the FLACC pain scale. Baseline information, including sex, age, operative side, procedures, and application of a tourniquet, was recorded and reviewed.

SPSS statistical package program (SPSS 19.0 version; SPSS Inc., Chicago, Illinois, USA) was used for statistical analysis. The categorical data were analyzed using the chi-square (*χ*^2^) test, and the continuous data were analyzed using Student’s *t* test. Fisher exact test was used under those circumstances with fewer subjects in groups of interest. Data were presented as mean ± SD (range), median (range), or *n* (%). A *P* value of less than 0.05 was considered significantly different.

## Result

As shown in Table 1, 150 patients (102/48, male/female) in the GA and 150 patients (104/46, male/female) in the GA + BPB group were included in our study. There existed no significant difference between the two groups regarding age, sex, fracture side, and types of procedures.

**Table 1:** Demographic and clinical parameters of children with elbow injuries

Parameters	GA (*n* = 150)	GA + BPB (*n* = 150)	*P* value
Age, years	7.2 ± 1.9	7.5 ± 1.7	0.152
Sex, male/female	102/48	104/46	0.803
Fracture side, L/R	95/55	92/58	0.721
LC	44 (29.3%)	45 (30.0%)	0.998
ME	23 (15.3%)	23 (15.3%)	
SC	60 (40.0%)	60 (40.0%)	
CV	23 (15.3%)	22 (14.7%)	

*GA* general anesthesia, *BPB* brachial plexus block, *LC* lateral condyle fracture, *ME* medial epicondyle fracture, *SC* supracondylar fracture, *CV* cubitus varus deformity

Data shown as mean ± SD or *n* (%)

As for the pain response after LCFH (Table 2), there were 44 patients (34/10, male/female) in the GA group and 45 patients (35/10, male/female) in the GA + BPB group. There existed no significant difference between the two groups concerning age, sex, fracture side, duration of surgery, and application of a tourniquet. The FLACC pain scale was significantly higher for those in the GA group (6.2 ± 0.8) when compared to the GA + BPB group (1.6 ± 0.5) (*P* < 0.001), and all patients in GA group were given additional analgesics in PACU. The pain response from the patient, caregiver, and HCW was significantly better in the GA + BPB group. The frequency of waking up from the sleep, calling nurse/doctor, and utilization of oral ibuprofen was significantly higher in the GA group than the GA + BPB group. Thirty-nine percent (17/44) of patients in the GA group required additional analgesics during the night shift, whereas only 8.9% (4/45) in the GA + BPB group required additional analgesics.

**Table 2:** Pain response in children with LC

Parameters		GA (*n* = 44)	GA + BPB (*n* = 45)	*P* value
Age, years		7.2 ± 1.8	7.2 ± 1.8	0.964
Sex, male/female		34/10	35/10	0.834
Fracture side, L/R		34/10	32/13	0.498
Duration of surgery, min		49.6 ± 6.7	48.9 ± 7.0	0.633
Tourniquet		0	0	> 0.999
FLACC in PACU		6.2 ± 0.8	1.6 ± 0.5	< 0.001^*^
Analgesic in PACU		44 (100%)	0	< 0.001^*^
FPS-R, patient		5.1 ± 1.0	3.4 ± 1.0	< 0.001^*^
NRS, caregiver		3.8 ± 0.8	2.3 ± 0.5	< 0.001^*^
FLACC in ward		3.1 ± 0.9	1.9 ± 0.5	< 0.001^*^
Wake from sleeping, times	0	0	27 (60.0%)	< 0.001^*^
	1	0	7 (15.6%)	
	2	10 (22.7%)	11 (24.4%)	
	≥ 3	34 (87.3%)	0	
Call nurse/doctor, times	0	9 (20.5%)	24 (53.3%)	< 0.001^*^
	1	6 (13.6%)	14 (31.1%)	
	2	16 (36.4%)	7 (15.6%)	
	≥ 3	13 (29.5%)	0	
Oral ibuprofen, times	0	0	45 (100%)	< 0.001^*^
	1	0	0	
	2	19 (43.2%)	0	
	≥ 3	25 (56.8%)	0	
Additional analgesic		17 (38.6%)	4 (8.9%)	< 0.001^*^

*PACU* postanesthesia care unit; *FLACC* Face, Leg, Activity, Cry, and Consolability; *FPS-R* Faces Pain Scale-Revised; *NRS* numeric rate scale

Data shown as mean ± SD or *n* (%)

* < 0.05

As for the pain response after MCFH (Table 3), there were 23 patients (18/5, male/female) in the GA group and 23 patients (18/5, male/female) in the GA + BPB group. There existed no significant difference between the two groups concerning age, sex, fracture side, duration of surgery, and application of a tourniquet. The FLACC pain scale was significantly higher for those in the GA group (6.0 ± 0.8) when compared to the GA + BPB group (1.5 ± 0.5) (*P* < 0.001), and all patients in the GA group required additional analgesics in PACU. The pain response from the patient, caregiver, and HCW was significantly better in the GA + BPB group. The frequency of waking up from the sleep, calling nurse/doctor, and utilization of oral ibuprofen was significantly higher in the GA than the GA + BPB. Fifty-two percent (12/23) of patients in the GA group required additional analgesics during the night shift, whereas only 13% (3/23) in the GA + BPB group required additional analgesics.

**Table 3:** Pain response in children with medial epicondyle fractures

Parameters		GA (*n* = 23)	GA + BPB (*n* = 23)	*P* value
Age, years		7.9 ± 1.2	8.5 ± 1.0	0.073
Sex, male/female		18/5	18/5	> 0.999
Fracture side, L/R		10/13	9/14	0.925
Duration of surgery, min		45.4 ± 8.3	43.9 ± 8.2	0.533
Tourniquet		0	0	> 0.99
FLACC in PACU		6.0 ± 0.8	1.5 ± 0.5	< 0.001^*^
Analgesic in PACU		23 (100%)	0	< 0.001^*^
FPS-R, patient		5.0 ± 1.0	3.1 ± 1.0	< 0.001^*^
NRS, caregiver		4.0 ± 0.8	2.5 ± 0.5	< 0.001^*^
FLACC in ward		3.2 ± 0.7	2.0 ± 0.6	< 0.001^*^
Wake from sleeping, times	0	0	10 (43.5%)	< 0.001^*^
	1	0	6 (26.1%)	
	2	6 (26.1%)	7 (30.4%)	
	≥ 3	17 (73.9%)	0	
Call nurse/doctor, times	0	1 (4.3%)	10 (43.5%)	0.002^*^
	1	9 (39.1%)	10 (43.5%)	
	2	10 (43.5%)	3 (13.0%)	
	≥ 3	3 (13.0%)	0	
Oral ibuprofen, times	0	0	23 (100%)	< 0.001^*^
	1	0	0	
	2	12 (52.2%)	0	
	≥ 3	11 (47.8%)	0	
Additional analgesic		12 (52.2%)	3 (13.0%)	0.005^*^

*PACU* postanesthesia care unit; *FLACC* Face, Leg, Activity, Cry, and Consolability; *FPS-R* Faces Pain Scale-Revised; *NRS* numeric rate scale

Data shown as mean ± SD or *n* (%)

* < 0.05

**Fig. 1:**
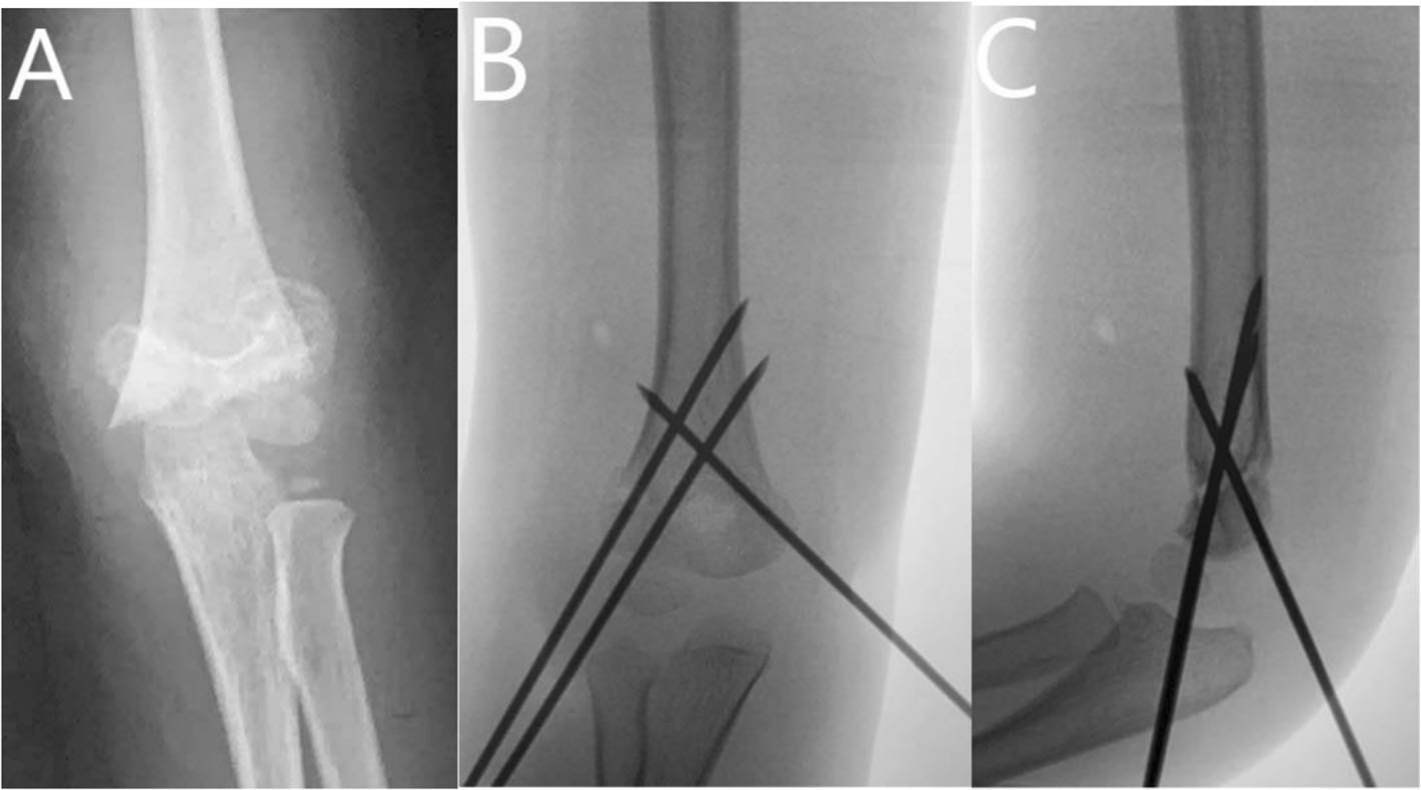
A 7-year boy of left supracondylar fracture treated with CRPP. **a** AP view of the elbow before the surgery. **b** AP view of the elbow after surgery. **c** Lateral view of the elbow after surgery

As for the pain response after SCFH (see Fig. 1, Table 4), there were 60 patients (38/22, male/female) in the GA group and 60 patients (36/24, male/female) in the GA + BPB group. There existed no significant difference between the two groups concerning age, sex, fracture side, duration of surgery, and application of a tourniquet. The FLACC pain scale was significantly higher for those in the GA group (6.0 ± 0.8) than the GA + BPB group (1.6 ± 0.5) (*P* < 0.001), and 75.0% (45/60) patients in GA group required additional analgesics in PACU. The pain response from the patient, caregiver, and HCW was significantly better in the GA + BPB group. The frequency of waking up from the sleep, calling nurse/doctor, and utilization of oral ibuprofen was significantly higher in the GA group than the GA + BPB group. Twenty-five percent (15/60) of patients in the GA group required additional analgesics during the night shift, whereas no patients in the GA + BPB group required additional analgesics.

**Table 4:** Pain response in children with supracondylar fractures

Parameters		GA (*n* = 60)	GA + BPB (*n* = 60)	*P* value
Age, years		6.4 ± 1.9	7.5 ± 1.7	0.002^*^
Sex, male/female		38/22	36/24	0.658
Fracture side, L/R		35/25	35/25	> 0.999
Duration of surgery, min		44.0 ± 9.2	43.5 ± 7.9	0.752
Tourniquet		0	0	> 0.999
FLACC in PACU		6.0 ± 0.8	1.6 ± 0.5	< 0.001^*^
Analgesic in PACU		45 (75.0%)	0	< 0.001^*^
FPS-R, patient		5.6 ± 1.1	3.4 ± 1.0	< 0.001^*^
NRS, caregiver		4.1 ± 0.8	2.4 ± 0.5	< 0.001^*^
FLACC in ward		3.1 ± 0.9	1.9 ± 0.6	< 0.001^*^
Wake from sleeping, times	0	0	28 (46.7%)	< 0.001^*^
	1	27 (45.0%)	21 (35.0%)	
	2	26 (43.3%)	11 (18.3%)	
	≥ 3	7 (11.7%)	0	
Call nurse/doctor, times	0	25 (41.7%)	36 (60.0%)	0.067
	1	17 (28.3%)	16 (26.7%)	
	2	15 (25.0%)	8 (13.3%)	
	≥ 3	3 (5.0%)	0	
Oral ibuprofen, times	0	0	60 (100%)	< 0.001^*^
	1	44 (73.3%)	0	
	2	16 (26.7%)	0	
	≥ 3	0	0	
Additional analgesic		15 (25.0%)	0	< 0.001^*^

*PACU* postanesthesia care unit; *FLACC* Face, Leg, Activity, Cry, and Consolability; *FPS-R* Faces Pain Scale-Revised; *NRS* numeric rate scale

Data shown as mean ± SD or *n* (%)

* < 0.05

As for the pain response after corrective osteotomy for cubitus varus deformity (Table 5), there were 23 patients (12/11, male/female) in the GA group and 22 patients (15/7, male/female) in the GA + BPB group. There existed no significant difference between the two groups concerning age, sex, fracture side, duration of surgery, and application of a tourniquet. The FLACC pain scale was significantly higher for those in the GA group (6.7 ± 0.7) when compared to the GA + BPB group (2.1 ± 0.7) (*P* < 0.001), and all patients in GA group required additional analgesics, whereas 54.5% (12/22) in the GA + BPB group required analgesics in PACU. The pain response from the patient, caregiver, and HCW was significantly better in the GA + BPB group. The frequency of waking up from the sleep, calling nurse/doctor, and utilization of oral ibuprofen was significantly higher in the GA group than the GA + BPB group. Seventy-four percent (17/23) of patients in the GA group required additional analgesics during the night shift, whereas only 27.3% (6/22) in the GA + BPB group required additional analgesics.

**Table 5:** Pain response in children with cubitus varus

Parameters		GA (*n* = 23)	GA + BPB (*n* = 22)	*P* value
Age, years		8.3 ± 1.4	6.8 ± 1.8	0.004^*^
Sex, male/female		12/11	15/7	0.128
Fracture side, L/R		16/7	16/6	0.843
Duration of surgery, min		58.6 ± 6.1	61.8 ± 6.3	0.097
Tourniquet		23 (100%)	22 (100%)	> 0.99
FLACC in PACU		6.7 ± 0.7	2.1 ± 0.7	< 0.001^*^
Analgesic in PACU		23 (100%)	12 (54.5%)	< 0.001^*^
FPS-R, patient		5.5 ± 0.8	3.9 ± 1.1	< 0.001^*^
NRS, caregiver		4.8 ± 0.5	2.7 ± 0.5	< 0.001^*^
FLACC in ward		3.9 ± 0.7	2.1 ± 0.7	< 0.001^*^
Wake from sleeping, times	0	0	3 (13.6%)	< 0.001^*^
	1	0	7 (31.8%)	
	2	4 (17.4%)	12 (54.5%)	
	≥ 3	19 (82.6%)	0	
Call nurse/doctor, times	0	2 (8.7%)	10 (45.5%)	0.002^*^
	1	7 (30.4%)	10 (45.5%)	
	2	8 (34.8%)	2 (9.1%)	
	≥ 3	6 (26.1%)	0	
Oral ibuprofen, times	0	0	4 (18.2%)	< 0.001^*^
	1	0	9 (40.9%)	
	2	6 (26.1%)	9 (40.9%)	
	≥ 3	17 (73.9%)	0	
Additional analgesic		17 (73.9%)	6 (27.3%)	< 0.001^*^

*PACU* postanesthesia care unit; *FLACC* Face, Leg, Activity, Cry, and Consolability; *FPS-R* Faces Pain Scale-Revised; *NRS* numeric rate scale

Data shown as mean ± SD or *n* (%)

* < 0.05

None of the patients reported BPB-related complications during the postoperative follow-up visit.

## Discussion

Additional BPB for elbow surgeries resulted in better postoperative pain response without significant BPB-related complications.

Elbow surgeries for fractures and cubitus varus deformity correction are common in the pediatric population [16, 17]. Although clinical outcomes of surgeries following fractures around the elbow are usually satisfactory, however, postoperative pain management remains challenging [2, 5, 6]. Pain is a significant parameter influencing recovery, early mobilization, and hospital stay [18]. BPB is an effective choice in the management of shoulder or humeral surgery in children [19–22]. Guidance with ultrasonography (US) improves the accuracy of needle advancement and anatomic identification of neural structures [23]. Although US-guided BPB is gaining popularity in the pediatric population [24, 25], the application of BPB in younger kids is still limited [26]. In our hospital, GA remains the preferred choice in pediatric surgeries. However, the results of this study indicated that the GA + BPB was more effective in reducing the pain following surgeries around the elbow.

In displaced LCFH and MCFH, open reduction and internal fixation (ORIF) is our preferred choice and usually yields satisfactory outcomes [27, 28]. Whereas, closed reduction and percutaneous pinning (CRPP) remains the primary choice for displaced SCFH [29]. Regardless of the fracture type and operative choice, the pain response was significantly lower in the GA + BPB group in our study. However, some patients receiving GA + BPB for corrective osteotomy and fixation for cubitus varus deformity complained of significant pain, possibly due to the application of a tourniquet.

Additionally, the cost for BPB is about 120 US dollars at our institute, and it is affordable for most patients and their families. There have been reports regarding the complications, including pneumothorax and neuropathy, related to the BPB [30, 31]; however, no BPB-related complications were apparent on the second postoperative day in our study.

An additional BPB is recommended for patients undergoing ORIF for elbow fractures; however, it might not be necessary for patients requiring CRPP only. More attention should be paid for postoperative pain management for patients undergoing corrective osteotomy and fixation with the application of a tourniquet for cubitus varus deformity. It is because the corrective osteotomy and fixation usually takes longer surgical time and prolonged use of the tourniquet that might result in tourniquet-related pain [32, 33].

There were certain limitations in our study. Firstly, it is a retrospective study with modest sample size; secondly, the cost of BPB is different in different countries, and cost-effective analysis remains to be investigated.

## Conclusion

An additional shot of BPB for patients undergoing surgeries for elbow surgeries resulted in better postoperative pain response in younger children without significant BPB-related complications. Those treated with GA + BPB had significantly less pain regardless of the fracture type. Patients treated with GA + BPB also woke up from sleep much less and utilized oral ibuprofen significantly less than those treated with GA alone regardless of fracture type.

## Data Availability

The datasets supporting the conclusion of this article are included within the article. Upon request, raw data can be provided by the corresponding author.

## Abbreviations

HCW: Healthcare worker
GA: General anesthesia
PACU: Postanesthesia care unit
BPB: Brachial plexus block
FLACC: Face, Leg, Activity, Cry, and Consolability pain scale
FPS-R: Faces Pain Scale-Revised
NRS: Numeric rate scale
ORIF: Open reduction and internal fixation
CRPP: Closed reduction and percutaneous pinning
